# Crystal Structures of the Carboxyl cGMP Binding Domain of the *Plasmodium falciparum* cGMP-dependent Protein Kinase Reveal a Novel Capping Triad Crucial for Merozoite Egress

**DOI:** 10.1371/journal.ppat.1004639

**Published:** 2015-02-03

**Authors:** Jeong Joo Kim, Christian Flueck, Eugen Franz, Eduardo Sanabria-Figueroa, Eloise Thompson, Robin Lorenz, Daniela Bertinetti, David A. Baker, Friedrich W. Herberg, Choel Kim

**Affiliations:** 1 Department of Pharmacology, Baylor College of Medicine, Houston, Texas, United States of America; 2 Department of Biochemistry, University of Kassel, Kassel, Hesse, Germany; 3 Faculty of Infectious and Tropical Diseases, London School of Hygiene & Tropical Medicine, London, United Kingdom; 4 Verna and Marrs McLean Department of Biochemistry and Molecular Biology, Baylor College of Medicine, Houston, Texas, United States of America; University of Geneva, SWITZERLAND

## Abstract

The *Plasmodium falciparum* cGMP-dependent protein kinase (*Pf*PKG) is a key regulator across the malaria parasite life cycle. Little is known about *Pf*PKG’s activation mechanism. Here we report that the carboxyl cyclic nucleotide binding domain functions as a “gatekeeper” for activation by providing the highest cGMP affinity and selectivity. To understand the mechanism, we have solved its crystal structures with and without cGMP at 2.0 and 1.9 Å, respectively. These structures revealed a *Pf*PKG-specific capping triad that forms upon cGMP binding, and disrupting the triad reduces kinase activity by 90%. Furthermore, mutating these residues in the parasite prevents blood stage merozoite egress, confirming the essential nature of the triad in the parasite. We propose a mechanism of activation where cGMP binding allosterically triggers the conformational change at the αC-helix, which bridges the regulatory and catalytic domains, causing the capping triad to form and stabilize the active conformation.

## Author Summary

Malaria causes up to a million fatalities per year worldwide. Most of these deaths are caused by *Plasmodium falciparum*, which has a complex life cycle in both humans and mosquitoes. One key regulator of this process is *P*. *falciparum* cGMP-dependent protein kinase (*Pf*PKG), the main effector of the cGMP-signaling pathway. Specifically blocking this kinase stops both replication and transmission of the parasites, suggesting that *Pf*PKG is a promising drug target. Here we identified the carboxyl cGMP-binding domain of *Pf*PKG serving as a gatekeeper for activation of the entire kinase by having the highest affinity and selectivity for cGMP. High-resolution crystal structures with and without cGMP allowed us to identify a novel cGMP capping triad that dynamically forms upon binding cGMP and stabilizes the activated conformation. Mutation of the capping triad forming residues not only reduces its kinase activity, but also prevents blood stage merozoite egress, demonstrating its crucial role in *Pf*PKG activation.

## Introduction

The malaria parasite *Plasmodium falciparum* has a complex life cycle comprising phases in both a human host and a mosquito vector [1–3]. Once the sporozoites are injected into a person by a mosquito during a blood meal, they must quickly relocate to the liver through the bloodstream and invade hepatocytes, where they then differentiate and generate thousands of liver stage merozoites within a single schizont. Following egress, the merozoites must then invade red blood cells (RBCs) and again multiply by asexual replication. A blood stage schizont is formed within 48 hours of red blood cell invasion and releases up to 32 new merozoites into the blood stream to invade fresh red cells. Proliferation of these asexual blood stage parasites leads to pathology, and furthermore, a small proportion of the asexual blood stage parasites commits to sexual development whereby distinct male and female gametocytes are formed, which are required for transmission to mosquitoes.

The complex malaria parasite life cycle is a highly regulated process, but the molecular details are poorly understood. Several biochemical and genetic studies indicate that cyclic nucleotides play a critical role at a number of stages in the *Plasmodium* life cycle [4,5]. Recent studies discovered that the *P*. *falciparum* cGMP-dependent protein kinase (*Pf*PKG) is involved in both asexual and sexual development [6–8] and specific inhibition of *Pf*PKG blocked progression of the life cycle at multiple stages [6,7,9].

**Fig 1:**
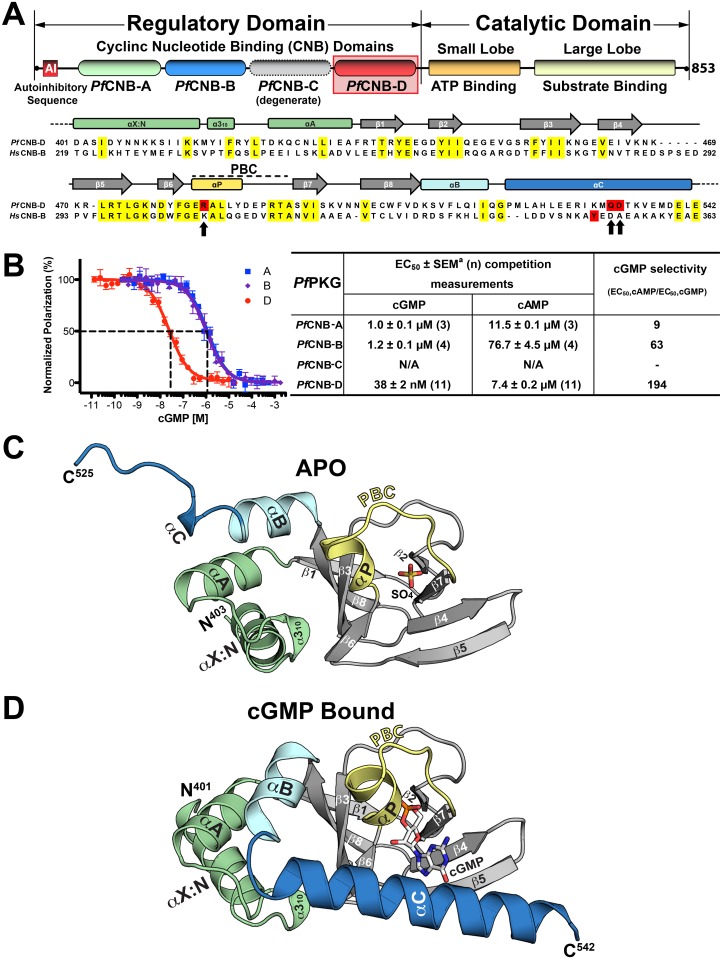
Domain organization and overall structures of *Pf*CNB-D. (A) Domain organization of *Pf*PKG and sequence alignment between *Pf*CNB-D and *Hs*CNB-B (Human PKG I). Identical residues are highlighted in yellow and the capping residues in both proteins are highlighted in red. The capping triad residues are also marked with arrows. (B) cGMP and cAMP affinities of *Pf*CNB domains. Competition FP curves for cGMP are shown on the left and EC_50_ values on the right. (C) Overall structure of *Pf*CNB-D without cGMP. The secondary structure elements are labeled. The phosphate binding cassette (PBC) is colored in yellow, the αB and αC helices in light cyan and blue, the N-terminal helices in light green and the β-barrel in gray. The N- and C-termini are labeled with their corresponding residue number seen in the final model. The sulfate ion co-crystallized with the protein is colored with its sulfur in yellow and oxygen in red. (D) Overall structure of the *Pf*CNB-D:cGMP complex. The structure is shown with the same color scheme as above except for cGMP. The cGMP is colored by atom type (carbon, white; nitrogen, blue; oxygen, red; and phosphorus, orange). All structure images were generated using *PyMOL* (Delano Scientific).

*P*. *falciparum* expresses a single PKG that has similar functional domains to mammalian PKG (mPKG), but exhibits some fundamental differences in domain organization. *Pf*PKG contains an N-terminal regulatory (R) domain including a possible auto-inhibitory (AI) sequence and a C-terminal catalytic (C) domain [10]. Both the R- and C-domains have approximately 30–40% sequence identity to their counterparts in mPKG. The major differences are that *Pf*PKG is larger than mPKG and does not contain an N-terminal dimerization domain, thus functioning as a monomer (Fig. 1A). Moreover, the R-domain of *Pf*PKG contains four consensus cyclic nucleotide-binding domains (*Pf*CNB-A, B, C and D), whereas mPKG has only two (mCNB-A and B) [11]. A previous study indicated that *Pf*CNB-C is degenerate since cGMP binding could not be detected [12]. The CNB domains commonly include an eight-stranded -barrel and a variable number of -helices [13]. Embedded within the -barrel, a key structural motif, the Phosphate Binding Cassette (PBC), binds the sugar-ribose portion of cGMP and consists of a short helix (P-helix) followed by a loop.

Cyclic GMP binding to *Pf*CNB-D has been shown to have the greatest effect on kinase activation [12]. In this study, each domain was disabled by mutation of a putative cGMP-binding residue and its effect on *Pf*PKG activation was analyzed. Disabling *Pf*CNB-D resulted in a 10-fold increase in the activation constant for cGMP (*Ka*_:cGMP_) and as much as a 50% reduction in maximal activity. In contrast, disabling *Pf*CNB-A, B, or C showed little to no effect. Since little structural information is available for mPKG and none for *Pf*PKG, the current model of activation is based on solution-based studies of mPKG [14,15]. The current model suggests that, in the absence of cGMP, the R-domain binds to the C-domain with high affinity, with the AI sequence bound within the catalytic cleft and preventing kinase activity. Cyclic GMP binding alters the conformation of the R-domain and releases the C-domain, causing activation. However, the details of the regulation and activation mechanisms are poorly understood. Here we report high-resolution crystal structures of *Pf*CNB-D in the presence and absence of cGMP. The data reveals not only the structural basis of cGMP selectivity, but also a *Pf*PKG-specific capping triad that allosterically triggers kinase activation. Mutagenesis of the capping triad residues impairs kinase activation and abrogates *P*. *falciparum* blood stage merozoite egress.

## Results

### *Pf*CNB-D binds cGMP with the highest affinity and selectivity

While all *Pf*PKG CNB domains have been shown to influence kinase activation to varying degrees [12], their individual affinities for either cGMP or cAMP have never been investigated. To assess their affinities, we purified each domain and measured their affinities for cGMP and cAMP using a competition fluorescence polarization (FP) assay. We expressed the CNB domains in *E*. *coli ∆cya TP2000*, which lacks adenylyl cyclase activity [16], hence avoiding bacterial cAMP contamination. Competition FP measurements show that the isolated *Pf*CNB-D domain binds cGMP with a half maximal effective concentration (EC_50_) of 38 nM, while the A and B domains bind cGMP with EC_50_ values ranging from 1 to 1.2 μM (Fig. 1B). In contrast, *Pf*CNB-D binds cAMP with an EC_50_ of 7.4 μM whereas other domains show values ranging from 12 to 77 μM. Thus, *Pf*CNB-D binds cGMP with the highest affinity and is over 190-fold selective for cGMP. *Pf*CNB-C shows very weak affinity for either 8-Fluo-cGMP or 8-Fluo-cAMP. This is expected because *Pf*CNB-C lacks the key cGMP interacting residues in its cGMP binding pocket (S1 Fig.) [10,12].

### Structure determination of *Pf*CNB-D in the apo and cGMP bound states and overall structures of *Pf*CNB-D

To understand the structural basis of its high affinity and selectivity for cGMP required for activation, we solved the structures of *Pf*CNB-D, both in the apo and cGMP bound states (Figs. 1C and 1D). The crystal structure of the apo protein was solved at 1.89 Å using the structure of CNB-B of human PKG Iα (residues 205–324) as a search model for molecular replacement (MR) (PDB code: 3SHR) [17]. The final model containing residues 403–526 shows four ordered sulfate ions, with one of the sulfates occupying the cyclic phosphate site due to a high sulfate concentration in the crystallizing condition. (Fig. 1C). The N-terminal portion of the αC-helix (residues 520–526) forms a loop, providing crystal contacts with the PBC of the neighboring molecule in the crystal lattice (S2 Fig.). The rest of the αC-helix (residues 527–542) is not modeled due to the lack of electron density (Fig. 1C).

The structure of the *Pf*CNB-D:cGMP complex was solved at 2.0 Å by MR using its apo structure as a search model. The final model of the complex includes residues 401–542. The entire structure shows robust electron density including the αC-helix (residues 520–542) (Fig. 1D). Additionally, the cGMP pocket shows clear electron density for cGMP bound in a *syn* conformation (S3 Fig.). While the overall structure of *Pf*CNB-D is similar to that of the highly cGMP selective human PKG I, the conformation of the C-terminal helix and the capping interaction at the cGMP pocket are strikingly different [18,19]. The structures of *Pf*CNB-D show a typical cyclic nucleotide binding domain fold consisting of an 8 stranded -barrel flanked by α helices at both termini [13,20]. Structural comparison of the apo and cGMP bound states also shows that the helical regions undergo major structural changes whereas the -barrel does not. The αC-helix is not ordered in the absence of cGMP, but becomes ordered upon binding cGMP and shields the cGMP pocket (Fig. 1). Statistics for crystallographic data and structural refinement are summarized in Table 1.

**Table 1:** Data and refinement statistics.

	Apo	cGMP bound
**Data collection**		
Wavelength (Å)	0.97931	0.97931
Space group	P2_1_2_1_2_1_	P3_1_21
Cell dimensions		
*a*, *b*, *c* (Å)	25.8, 57.7, 82.8	67.3, 67.3, 59.5
α, β, γ (°)	90, 90, 90	90, 90, 120
Resolution (Å)	33.6–1.89	26.2–2.00
R_sym_ or R_merge_	8.8 (48.4)*	8.9 (29.7)
I/σI	18.2 (6.0)	18.0 (7.5)
Completeness (%)	99.8 (100)	99.2 (98.0)
Redundancy	6.9 (7.1)	10.9 (11.0)
**Refinement**		
Resolution (Å)	27.2–1.89	26.2–2.00
No. reflections	10438	10665
R_work_/R_free_ ^†^	18.25/22.85	16.82/20.50
No. atoms		
Proteins	1045	1193
Ligand/ion	20	119
Water	51	50
B-factors		
Protein	28.8	29.1
Ligand/ion	43.8	43.3
Water	34.1	37.2
R.m.s. deviations		
Bond lengths (Å)	0.007	0.008
Bond angles (°)	1.075	1.136

*Highest resolution shell is shown in parenthesis.

^†^ 5.0% of the observed intensities was excluded from refinement for cross validation purposes.

### The *Pf*CNB-D:cGMP complex shares many structural features with human PKG I, but shows a distinct capping mechanism.

**Fig 2:**
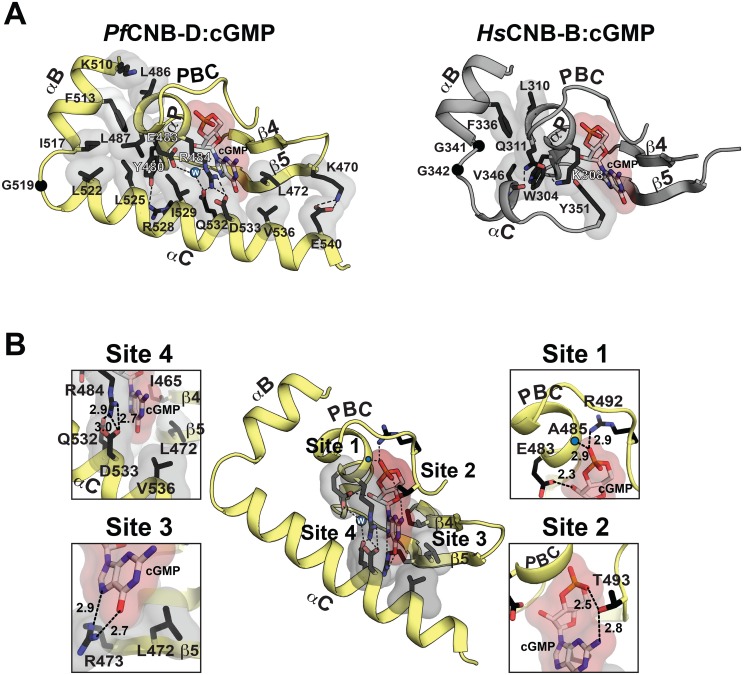
Structural comparison between *Pf*CNB-D and CNB-B and cGMP binding pocket of *Pf*CNB-D. (A) The cGMP pockets *Pf*CNB-D and CNB-B from human PKG Iβ (PDB code: 4KU7) are shown. The cGMP pocket of *Pf*CNB-D is colored in yellow (left) and the pocket of PKG Iβ CNB-B in gray (right). Key residues that stabilize the C-helix including the capping residues are shown with transparent surface in the following color theme: side chain carbon, black; oxygen, red; nitrogen. A water molecule captured between E483, R484, and Q532 is shown as a blue sphere. The C atoms of glycine residues located between at the αB and αC helices are shown as black spheres. Hydrogen bonds are shown as dotted lines. (B) Detailed interactions between *Pf*CNB-D and cGMP. Zoomed in views for each cGMP binding site are shown on either side. The backbone amide of A485 is marked with a blue dot. The individual cGMP interacting residues are shown with the following color theme: side chain carbon, black; oxygen, red; nitrogen, blue. The residues binds cGMP with VDW contacts including the capping residues are shown with transparent surface. Hydrogen bonds are shown as dotted lines with their distances in Å units.

The crystal structure of the *Pf*CNB-D:cGMP complex shows that, while the overall structure of its cGMP pocket is similar to that of the human PKG I CNB-B, its capping mechanism is completely different and the pocket is highly shielded from solvent due to the fully ordered C-helix (Fig. 2A) [18,19]. The cGMP pocket can be broken into four sites, and interactions at the first three sites are similar to those seen in the human PKG I CNB-B:cGMP complex (Figs. 2A and 2B). These include E483 and A485, which capture the sugar-phosphate of cGMP through hydrogen bonds (Site 1), T493, which bridges the O2A of cyclic phosphate and the N2 of guanine though hydrogen bonding interactions (Site 2), and L472 and R473 at strand 5, which specifically recognize the guanine moiety through Van der Waals (VDW) interaction and hydrogen bonds (Site 3).

Site 4 is distinct from mPKG and consists of R484 at the PBC and Q532/D533 on the αC-helix, which form a unique capping interaction with cGMP. As seen in Fig. 2B, the side chains of R484 and Q532 form a hydrogen bond and stack against the guanine moiety of cGMP, shielding the entire cGMP through VDW contacts. In particular, the hydrophobic arm of R484 shields the sugar-phosphate whereas its guanidinium group along with the side chain of Q532 shields the guanine moiety, providing a hydrophobic cap for cGMP. Additionally, R484 forms a salt bridge with D533. The guanine moiety of cGMP becomes sandwiched between the R484/Q532 and I465 at strand 4 (Fig. 2B). Moreover, an ordered water molecule captured between E483, R484, and Q532 interlinks these residues, further stabilizing the capping interaction (Fig. 2B). R484 of *Pf*PKG corresponds to K308 in human PKG I (Figs. 2A and 2B). However, K308 forms no interaction with the αC-helix or the bound cGMP, but instead interacts with a neighboring Q311 through a hydrogen bond (Fig. 2A). Despite little sequence similarity at the αC helix, the side chain of Q532 in *Pf*PKG aligns structurally with that of the aromatic side chain of Y351, the conserved capping residue in human PKG Iβ, further confirming its role as a capping residue for cGMP (Figs. 1A and 2A). Finally, an additional αC-helix residue, V536, interacts with L472 at strand 5 through a VDW contact and shields the guanine moiety from solvent (Fig. 2A).

### The unique capping interaction is essential in cGMP binding and kinase activation

To test the role of the capping triad residues in cGMP binding, we mutated these residues to alanine both in *Pf*CNB-D and in the full length R-domain and measured their affinities for cGMP and cAMP using a competition FP assay (Table 2). Our measurements showed that mutating any of the capping triad residues drastically reduced its affinity for cGMP, but only showed a slight reduction in its cAMP affinity. Specifically, in the truncated *Pf*CNB-D, mutating the capping triad increases its EC_50_ values for cGMP from 38 nM to 7 μM whereas the same mutations increase the values for cAMP from 7.4 μM to 65 μM. In the full length R-domain containing all four CNBs, the same set of mutations increases its EC_50_ values for cGMP from 79 nM to as much as 1.4 μM, compared with the cAMP values changing from 9.6 μM to 21 μM further demonstrating the important role of the capping triad in high affinity cGMP binding.

**Table 2:** Cyclic nucleotide binding affinities of *Pf*PKG wild type and mutants.

Constructs	EC_50_ ± SEM *(n) competition experiments	
	cGMP	cAMP
***Pf*PKG CNB-D**_(401–542)_		
Wild type	37.8 ± 1.8 nM (11)	7.4 ± 0.2 μM (11)
R484A	3.2 ± 0.4 μM (8)	43.5 ± 3.1 μM (8)
Q532A	2.4 ± 0.1 μM (7)	60.8 ± 1.9 μM (7)
D533A	760 ± 40 nM (7)	37.3 ± 1.8 μM (7)
R484A/Q532A/D533A	7.2 ± 0.3 μM (7)	65.1 ± 3.2 μM (7)
***Pf*PKG full regulatory domain**_(1–542)_		
Wild type	79.0 ± 4.0 nM (10)	9.6 ± 0.8 μM (7)
R484A	960 ± 20 nM (7)	19.0 ± 2.1 μM (6)
Q532A	2.2 ± 0.1 μM (6)	48.1 ± 2.3 μM (6)
D533A	610 ± 50 nM (6)	22.5 ± 3.3 μM (6)
R484A/Q532A/D533A	1.4 ± 0.1 μM (9)	21.0 ± 1.4 μM (7)

* FP measurements were at least in duplicate. EC_50_ is the half-maximal effective concentration, and SEM is standard error of mean.

**Fig 3:**
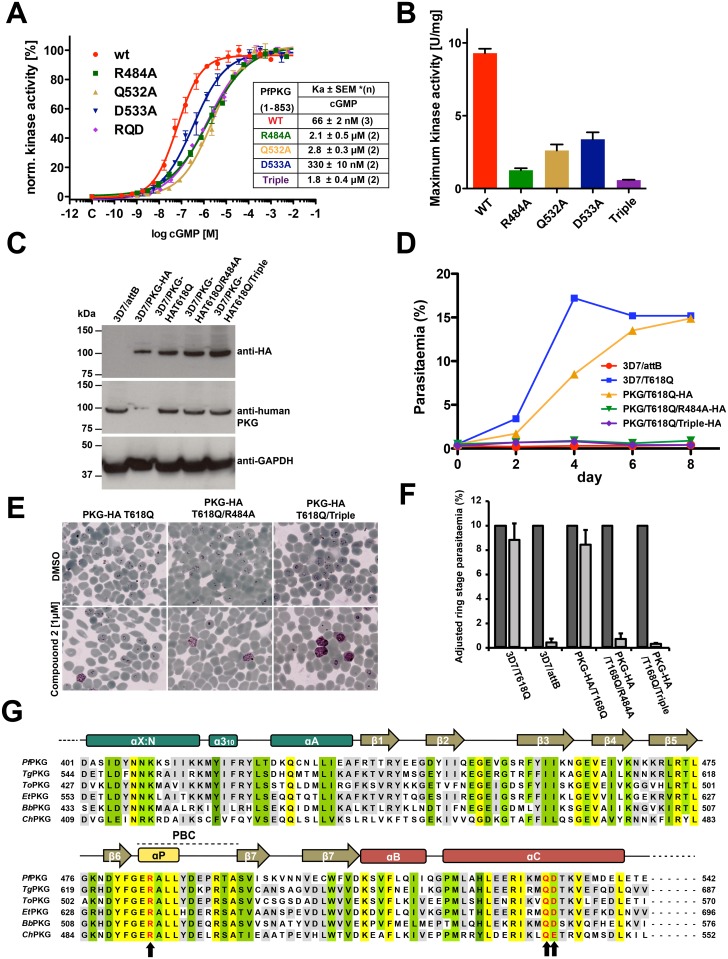
Role of the unique capping triad at *Pf*CNB-D in *Pf*PKG activation and *Plasmodium* parasite life cycle. (A) Role of the capping motif forming residues in kinase activation. Individual curves with error bars denoting standard error of mean are shown on the left and corresponding *Ka* values for WT and capping triad mutants are shown on the right. Each data curve was normalized by designating the lowest value of the data set as 0% and the highest value as 100%. (B) The specific activities of the WT and mutants at 10 μM cGMP are shown as bar graphs with error bars denoting standard error of mean. (C) Immunoblot showing co-expression of endogenous *Pf*PKG and ectopic *Pf*PKG-HA in transgenic schizonts. 3D7/attB is the parental line and 3D7/PKG-HA is a previously established line where the endogenous *Pf*PKG gene has been HA-tagged. Blots were incubated with anti-HA, anti-human PKG, and anti-*Pf*GAPDH as a loading control. Note that the anti-human PKG antibody does not react with HA-tagged PKG (lane 2) because the free carboxyl terminus is crucial for antibody binding. This allows the differentiation between endogenous and HA-tagged PKG. (D) Growth of the transgenic lines in the presence of 1 μM compound 2 over 8 days. The established compound 2-resistant line 3D7/T618Q was included as a positive control. (E) Late trophozoites/early schizonts of the three transgenics were cultured for 12 hours in the presence of 1 μM compound 2 (lower panels) or DMSO (upper panels) and parasite development examined on Giemsa-stained blood smears. (F) Quantification of *(E)*. >1000 cells were counted for each culture and condition and ring stage parasitaemia determined. Data represent the mean of three experiments (error bars = SD). Dark grey bars are DMSO controls, light grey bars 1 μM compound 2 treated samples. For each sample, parasitaemia was adjusted to make the DMSO control 10% to eliminate variability from differences in parasitaemia between experiments. (G) Sequence alignment of apicomplexan PKGs. Conserved residues are shaded in yellow (identical), in green (functionally similar), and in gray (identical in >66%). The capping triad residues are typed in red and marked with arrows. *Toxoplasma gondii* PKG, TgPKG; *Theileria orientalis* PKG, ToPKG; *Eimeria tenella* PKG, EtPKG; *Babesia bovis* PKG, BbPKG, and *Cryptosporidium hominis*, ChPKG.

Next, we generated the same set of mutations in full-length *Pf*PKG (residues 1–853) (S6 Fig.) and measured its effect on kinase activation using a microfluidic mobility-shift assay (Figs. 3A and 3B). Singly mutating R484 or Q532 to alanine increased the activation constants for cGMP (*K*_*a*:***c***GMP_) from 66 nM to 2–3 μM, whereas mutating D533 showed only a slight increase (to 330 nM) (S1 and S2 Tables). In addition, mutating all three residues increased its *K*_*a*:***c***GMP_ over 28-fold (to >1.8 μM) and reduced its maximum activity by 90% (Figs. 3A and 3B). In summary, our data demonstrate that the capping triad is crucial not only for cGMP binding, but also for cGMP-dependent activation of *Pf*PKG.

### Mutating the capping triad residues disables merozoite egress in *P*. *falciparum*

To determine the role of the capping triad on malaria parasite development, we utilized a chemical genetic approach employing a selective PKG inhibitor (compound 2, an imidazopyridine) and a transgenic parasite line expressing an engineered PKG allele (incorporating a threonine to glutamine substitution, T618Q) that confers inhibitor resistance [6–8,21]. While compound 2 inhibits endogenous *Pf*PKG by occupying a hydrophobic pocket adjoining the ATP-binding domain, the T618Q mutant has a bulkier side chain that prevents inhibitor binding and is no longer sensitive to compound 2 retaining kinase activity.

In this study, we generated three new *P*. *falciparum* transgenic lines containing an ectopic copy of the full length HA-tagged *pkg* gene harboring the T618Q mutation, but also containing either the R484A mutation (PKG_T618Q/R484A_-3xHA), the R484A/Q532A/D533A triple mutation (PKG_T618Q/R484A/Q532A/D533A_-3xHA) or no additional mutation (PKG_T618Q_-3xHA) as a control. The HA-tagged pkg genes were integrated as a single copy into a modified pseudogene locus via Bxb1 integrase mediated attB/attP recombination (S7 Fig.)[22]. Expression of the ectopic copy is driven by the parasite-derived *ama1* promoter displaying a schizont-specific activity profile similar to the endogenous *pkg* promoter. In the absence of compound 2, these lines all express two functional copies of the *Pf*PKG enzyme and expression of HA-tagged PKG is comparable between the three lines (Fig. 3C). All transgenic lines grow at equivalent rates to the parental line (3D7/attB) and an additional untagged control line (*Pf*PKG_T618Q_), in which the gatekeeper mutation was previously introduced by allelic replacement (S8 Fig.) [6]. These controls confirm that over-expression of *Pf*PKG has no effect on growth rate and that expression of the capping triad mutants has no dominant negative effect (S8 Fig.). Upon addition of compound 2, the endogenous *Pf*PKG is fully inhibited, allowing us to measure the effects on parasite development of the mutant *Pf*PKGs and to test whether they could rescue the chemically inhibited endogenous enzyme (S9 Fig.). Growth of the three transgenic lines in the presence of compound 2 (1 μM) was compared to that of the parental line and the original untagged T618Q allelic replacement control line. *Pf*PKG_T618Q/R484A_-3xHA and *Pf*PKG_T618Q/R484A/Q532A/D533A_-3xHA transgenics did not proliferate for the duration of the experiment (7 days) whereas the *Pf*PKG_T618Q_-3xHA control was able to grow at a similar rate to the allelic replacement control line of *Pf*PKG_T618Q_ (Fig. 3D). Examination of Giemsa-stained parasites in blood films showed that transgenic lines with the capping triad mutations, accumulated at the schizont stage and did not progress to ring stage parasites that result from merozoite egress and invasion of new erythrocytes in the presence of compound 2 (Fig. 3E). By contrast, high levels of ring stage parasites were observed in untreated cultures, the *Pf*PKG_T618Q_ replacement line and the *Pf*PKG_T618Q_-3xHA control line. This block in parasite development was examined in more detail by flow cytometry and the results confirmed the requirement of an inhibitor-resistant *Pf*PKG to progress from schizont to ring stage parasites (Fig. 3F). Taken together, the results demonstrate that mutating the capping residues impairs blood stage egress and reinvasion. Moreover, the results are consistent with our previous findings that *Pf*PKG activity is essential for merozoite egress and subsequent invasion of erythrocytes and confirms that the capping triad is required for *Pf*PKG activity *in vivo*.

## Discussion

### *Pf*CNB-D functions as a “gatekeeper” for activation of *Pf*PKG

Our study of *Pf*CNB-D has demonstrated its role as a “gatekeeper” domain for cGMP-dependent activation. Since cGMP binding is the first step in activation, it is essential to understand how cGMP binds the R-domain and what structural changes are associated with this binding event (see below). While there have been several studies reporting binding affinities of mPKG [18,23,24], the binding affinities for *Pf*PKG prior to this study were unknown. Previous mutagenesis studies of *Pf*PKG and its orthologue from *Eimeria tenella* (*Et*PKG) showed that, while all CNB domains are required for maximal activation, disabling the CNB-D domain had the largest effect on activation [12,25]. Consistent with these studies, we demonstrate that the largest effect on activation is due to *Pf*CNB-D having the highest affinity and selectivity for cGMP (Fig. 1B). Additionally, since cGMP binding to all three functional CNB domains is required for maximum kinase activity [12,25], the high selectivity of *Pf*CNB-D for cGMP would limit activation by cAMP, and facilitate full activation selectively by cGMP. Therefore, we suggest that *Pf*CNB-D serves as “gatekeeper” for *Pf*PKG activation.

### Stepwise transition from the apo to the cGMP bound state.

**Fig 4:**
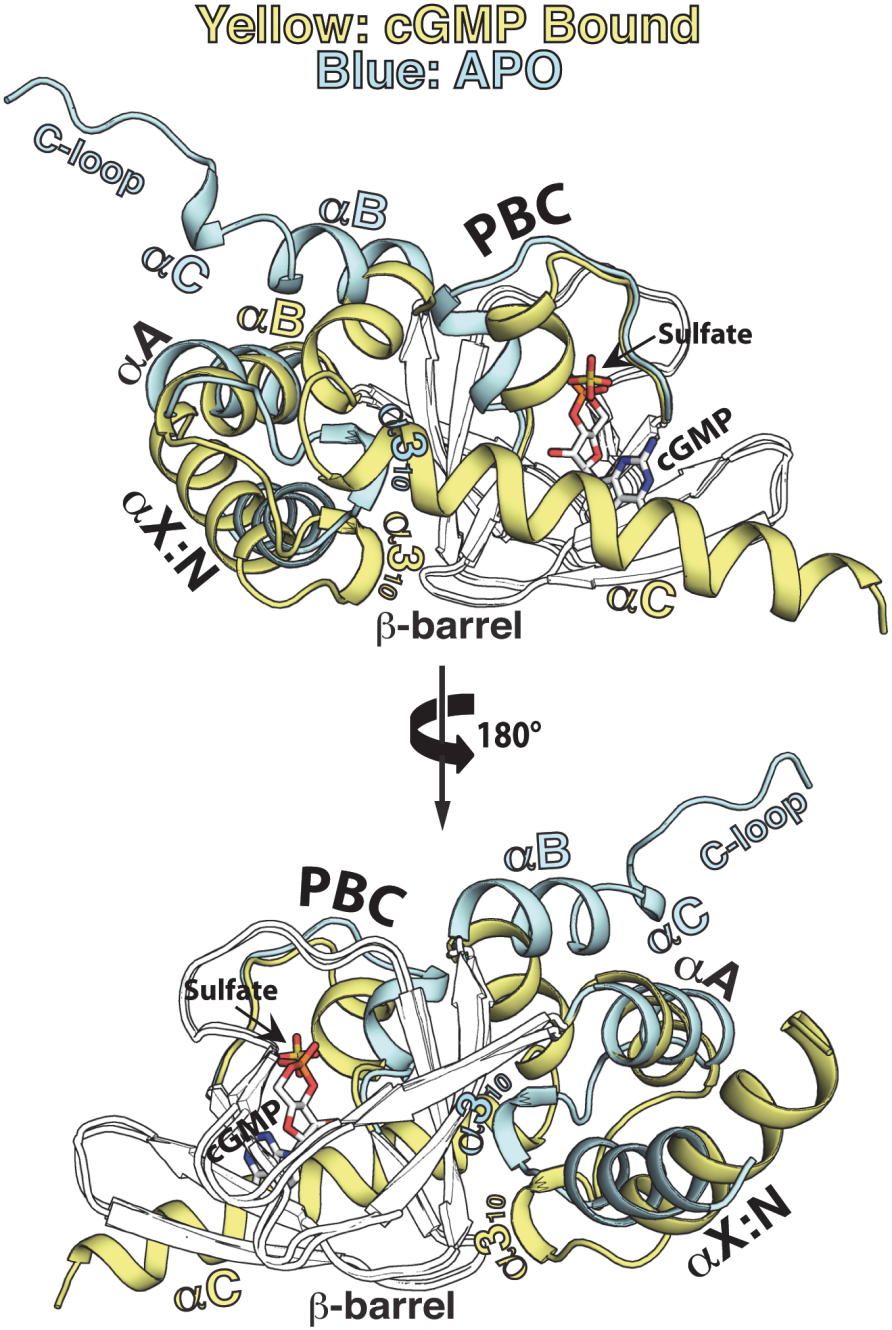
Structural comparison between the apo- and cGMP bound *Pf*CNB-D. The apo and *Pf*CNB-D:cGMP complex structures are aligned at the β-barrel region (not colored). The helical subdomain of the apo structure is colored in light cyan and that of the cGMP complex structure in yellow.

**Fig 5:**
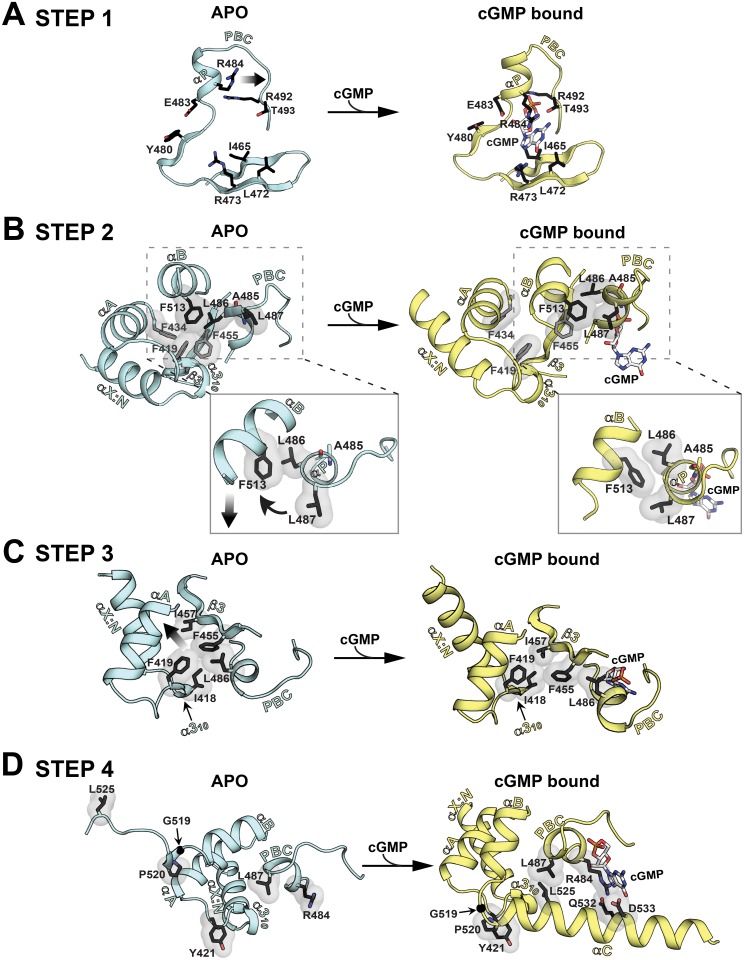
Conformational changes upon cGMP binding. Conformational changes are depicted in a step-wise fashion. The hydrophobic core, hinge, and cap forming residues are shown with transparent surface. Their side chain carbons are colored in black, oxygen in red, and nitrogen in blue.. (A) Step 1: PBC assuming a closed conformation upon cGMP binding. (B) Step 2: A cogwheel-like motion between the αP- and αB-helices allowing the αB-helix to move toward the PBC. Zoomed in views highlight changes in hydrophobic interactions. (C) Step 3: The N3A motif moving away from the PBC. (D) Step 4: The αC-helix shielding the bottom of the cGMP pocket and enabling the capping triad formation.

Structural comparison of the apo and cGMP bound states reveals two distinct sets of hydrophobic networks that link the PBC to the rest of the CNB domain. Careful analysis suggests that these networks transmit cGMP-induced changes at the PBC to the rest of the helical regions and allow *Pf*CNB-D to transition from one state to the other (Figs. 1, 4 and 5). In the absence of cGMP, the PBC is open with its αP-helix positioned away from the -barrel. The αB-helix, which interacts directly with the PBC, is tilted away from the -barrel (Figs. 4 and 5A). The α3_10_-helix of the N3A motif interacts directly with the PBC (Figs. 5B and S4A). Three phenylalanines from different regions, F419 (α3_10_-helix), F434 (αA-helix) and F455 (3), together with the hinge residues, L486 (αP-helix) and F513 (αB-helix) as previously defined in the CNB domain of Epac2, form a hydrophobic core that stabilizes the apo conformation [26].

Upon cGMP binding, the structural changes occur at the helical regions in a highly coordinated manner. The first step is the αP-helix adopting a compact conformation when cGMP binds to the PBC (Figs. 5A and S4B). This structural change is driven by the hydrogen bond formed between O1A of the cyclic phosphate and the backbone amide of A485, which is seen in other CNBs (Fig. S4B) [20]. This causes the αP-helix to rotate slightly clock-wise and tilt approximately 20° toward the -barrel (Figs. 5A and S4B). The second step involves L486 and L487 on the αP-helix, which together with F513 on the αB-helix, undergo a cogwheel-like motion causing the αB-helix to move toward the PBC (Fig. 5B). The third step is the N3A motif moving away from the PBC, pushed away by the αB-helix moving toward the PBC (Fig. 5C). These changes occur because the hydrophobic tip of the α3_10_-helix (I418 and F419) no longer interacts with L486 at the PBC, but slides along strand β3 and forms new interactions with I457. Finally, the αC-helix moves close to the cGMP pocket and forms the capping triad shielding the bound cGMP (Fig. 5D). One side of the αC helix displays several residues that form a Velcro-like interaction with the rest of the domain and the bound cGMP (Fig. 2A). An extensive network formed between two interlinking loops including captured waters (the loops between the α3_10_-αA helices and the αB-αC helices) helps form this Velcro-like interaction (S5 Fig.). In particular, a hydrophobic interaction between P520 (at αC) and Y421 (at the α3_10_ and αA loop) stabilizes the flexible αB-αC loop with G519 and orients the N-terminal region of the αC-helix for the Velcro-like interaction (Figs. 5D and S5).

### Structural basis of cGMP selectivity

Structural comparison of *Pf*CNB-D with the cGMP selective human PKG Iβ CNB-B combined with the sequence comparison with the other *Pf*CNB domains confirms the amino acids required for cGMP selectivity and explains the different cyclic nucleotide affinity values measured for *Pf*CNB domains. Both structures show highly similar interactions with cGMP including charged interactions at the PBC and β5 that specifically recognize the guanine moiety (Sites 1–3) and hydrophobic interactions that shield the bound cGMP, although the nature of the latter interactions differs (Fig. 2A). Due to the capping triad and V546-L472 interactions at Site 4, the cGMP pocket of *Pf*CNB-D is more shielded compared to the human PKG Iβ CNB-B. Since these are not charged interactions specific for cGMP, the more shielded pocket could result in higher affinity for both cGMP and cAMP. Consistent with the structure, our measurements on *Pf*CNB-D show smaller EC_50_ values for both cGMP and cAMP compared to these of human PKG Iβ CNB-B (EC_50 cGMP_ = 215 nM and EC_50 cAMP_ = 52 μM) [18]. Sequence alignment shows that *Pf*CNB- A and B have either the same or chemically similar residues that can recognize cGMP at the first three sites whereas *Pf*CNB-C lacks most of these residues (S1 Fig.). These observations are also consistent with our measurements showing clear cGMP binding in *Pf*CNB-A and B, and little to no binding in *Pf*CNB-C. Sequence alignment also shows that *Pf*CNB-B has an arginine analogous to the capping triad forming R484 of *Pf*CNB-D, but lacks the analogous Q532/D533 residues at the corresponding positions in the C-terminus suggesting that the capping triad is unlikely to form in *Pf*CNB-B. Consistent with these observations, mutating the capping triad residues to alanine in *Pf*CNB-D reduces its cGMP affinity to a similar level as in *Pf*CNB-A and B.

### Role of the αC-helix and capping triad in activation

While similar conformational changes were observed in the helical subdomains of the human PKG Iβ CNB-B, these changes at the αC-helix and the capping mechanism are very different between the two structures [18,19]. The αC-helix in the human PKG Iβ CNB-B:cGMP complex shows a single turn of helix followed by a short loop and only shields the bound cGMP through the capping interaction, whereas the fully helical αC helix of *Pf*CNB-D is more ordered and shields the entire base of the β barrel through a Velcro-like interaction. Moreover, while a single aromatic residue at the αC-helix provides the capping interaction in human PKG Iβ, the triad formed by R484/Q532/D533 provides the capping interaction in *Pf*PKG. Although these structures only represent a small portion of the R-domain and may suffer from artifacts associated with using truncated domains for crystallization and having different crystal packing environments, the dramatic conformational changes at the αC-helix are observed in both structures suggesting its highly dynamic nature. Since the activity of PKG is allosterically regulated by the structural rearrangement within its R-domain upon cGMP binding and the αC-helix is located between the R- and C- domains [14,20], we hypothesized that the conformational change of the αC-helix is a key step in disrupting the high affinity R-C interaction and releasing the C-domain and that the capping triad is a key structural feature that stabilizes the active conformation.

Our binding and activation results strongly support this hypothesis. Reductions in cGMP binding affinity were seen for all individual mutants, R484A, Q532A, or D533A of *Pf*CNB-D and the full-length R domain, with D533A having the least reduction. The same trend is observed for kinase activation where major reductions were seen in R484A or Q532A as expected, but only a slight reduction in D533A. Mutating all three showed the highest reduction both in cGMP affinity and in activity compared to wild type. These results suggest that the interaction between R484 and Q532 plays a bigger role in cGMP binding and activation than the R484-D533 interaction. Possibly, this is because Q532 is located on the inner side of the αC-helix and is directly involved in the capping interaction with cGMP. In contrast, D533 is located on the outer surface of the αC-helix with its side chain exposed to solvent contributing much less to the capping interaction.

Lastly, we show that mutation of the capping triad in the parasite itself blocks blood stage development. *Pf*PKG plays an essential role in egress of blood stage merozoites from erythrocytes [7], and inhibition of *Pf*PKG prevents the release of proteins from apical organelles required for egress and reinvasion [8]. It has also been suggested that *Pf*PKG is the universal regulator of calcium release across the malaria parasite life cycle, which is achieved by phosphorylation of phospholipid kinases and generation of IP_3_ [21]. Disruption of PKG function, therefore, interferes with a number of essential downstream signaling processes. Here we used a chemical-genetic approach to demonstrate that disruption of the capping triad within the highly cGMP selective *Pf*CNB-D domain, abrogates PKG’s ability to promote blood-stage egress, reinvasion, and signaling through downstream effectors, further strengthening the conclusion that the capping triad formation is an integral part of the activation mechanism. Thus, we propose that the αC-helix serves as an allosteric switch that triggers activation upon cGMP binding. Additionally, the αC-helix, strategically located between the R and C domains functioning as a molecular switch for activation, is likely to be a common feature of all PKGs, though the molecular details of the capping may differ due to the highly divergent amino acid sequence in this region. Amino acid sequence comparisons with other apicomplexan parasitic PKGs strongly suggest that the capping triad is a shared feature that could be targeted for developing broad-spectrum inhibitors against this family of important parasites (Fig. 3G) affecting humans and animals, that includes *Toxoplasma*, *Cryptosporidium*, and *Eimeria*. [10,27]. We envision such compounds that specifically bind the *Pf*CNB-D pocket and disrupt the formation of the capping triad will be potent inhibitors of these parasites.

## Methods

### Construct design, protein expression and purification

A manually designed construct consisting of *Pf*PKG amino acids 1–853 with codons optimized for expression in *E*.*coli* was synthesized at Life Technologies. All *Pf*PKG constructs were ligated into the His-tagged bacterial expression vector, pQTEV [28]. In designing the expression constructs of each CNB, we used the sequence of the cGMP selective CNB-B that we recently reported [18]. The domain boundaries for the individual constructs are: residues 1–173 for CNB-A, 158–294 for CNB-B, 275–428 for CNB-C and 401–542 for CNB-D. All *Pf*PKG proteins were expressed in TP2000 *E*. *coli*. The cells were grown at 37˚C to an OD_600nm_ of 0.6, then induced with 0.5 mM IPTG, and grown for an additional 10 hours at 25˚C. Cells were harvested by centrifugation, then resuspended in 50 mM potassium phosphate (pH 7.5), 500 mM NaCl and 1 mM β-mercaptoethanol, and lysed using a cell disruptor (Constant Systems).

The lysate was cleared using ultracentrifugation and the supernatant was loaded onto an IMAC nickel column (Bio-Rad) on ÄKTA purifier (GE Healthcare). The N-terminal his-tagged *Pf*PKGs were eluted by linear gradient with cell suspension buffer containing 300 mM imidazole. To remove the His-tag, the sample was incubated with His-tagged tobacco etch virus (TEV) protease in a 50:1 molar ratio at 4˚C overnight and loaded onto Bio-Rad IMAC column for TEV separation. The sample without his-tag was obtained by collecting flow-through fractions. The sample was loaded onto a HiLoad 16/60 Superdex 75 gel filtration column (GE healthcare) equilibrated with a buffer containing 25 mM Tris (pH 7.5), 150 mM NaCl, and 1 mM TCEP. For the purification of wild type and mutant full-length *Pf*PKG, we performed an additional chromatography step using a Resource Q anion exchange column (GE Healthcare) before the final size exclusion step using a Hiload 16/60 Superdex 200 column (GE Healthcare).

### Crystallization, data collection, phasing, model building, and refinement

To obtain crystals of the *Pf*CNB-D:cGMP complex, the protein sample was pre-incubated with 5 mM cGMP and concentrated to 50 mg/mL using a 10 kDa cutoff Amicon Ultra (Millipore). Crystals were obtained using the hanging drop method. 2 μL of protein solution was mixed with 2 μL of reservoir solution containing 0.2 M lithium sulfate, 15% ethanol, 0.1 M citrate at pH 5.5 and 10% 1,5-diaminopentane dihydrochloride. After three days incubation at 4˚C, drops produced rod-shape crystals belonging to a P3_1_21 space group that diffracted to 2.0 Å resolution. Apo crystals were obtained at 22˚C in a solution containing 0.2 M lithium sulfate, 25% (w/v) PEG 3350 and 0.1 M Bis-Tris (pH 5.5). The apo crystal belongs to a P2_1_2_1_2_1_ space group and diffracted to 1.89 Å resolution. All protein concentrations were measured using Bradford assay. Crystals of the cGMP complex and the apo form were cryoprotected with 25% ethylene glycol and Paratone-N, respectively, before freezing.

Diffraction experiments were performed at the Advanced Photon Source (Argonne, IL, USA). Diffraction data were processed and scaled using iMosflm [29] with satisfactory statistics (Table 1). Phasing of the apo structure was accomplished using the molecular replacement program Phaser-MR [30] with the previously determined C-terminal cGMP binding domain of PKG Iα structure (PDB code: 3SHR; residues 205–324) as a search model. Manual building of the model was performed using the program *Coot* [31], followed by rounds of refinement using phenix.refine [32]. Phasing of the *Pf*CNB-D:cGMP structure was accomplished using the molecular replacement program Phaser-MR [30] using a truncated apo structure of *Pf*CNB-D (residues 404–518) as a search model. Manual building of the model was also performed using *Coot* [31], followed by rounds of refinement using phenix.refine [32]. The final refinement round included restrained TLS refinement parameters [33].

### Fluorescence polarization

The direct fluorescence polarization (FP) assay was performed following the procedure from Moll et al [34]. Measurements were performed in 150 mM NaCl, 20 mM MOPS plus 0.005% (w/v) CHAPS pH 7.0 using the Fusion a-FP microtiter plate reader at room temperature in a 384 well microtiterplate (Perkin Elmer, Optiplate, black). The protein concentration was varied while the concentration of 8-Fluo-cAMP/8-Fluo-cGMP (Biolog Life Science Institute (Bremen, Germany)) was fixed at 1–5 nM. The FP signal was detected for 2 seconds at Ex 485 nm and Em 535 nm with a PMT Voltage of 1.100 V. Data were analyzed with GraphPad Prism 5.03 (GraphPad Software, San Diego, CA) by plotting the polarization signal in mPol against the logarithm of the protein concentration. The K_D_ values were calculated from sigmoidal dose-response curves.

For competition FP experiments, the protein concentration and the concentration of 8-Fluo-cGMP were fixed to give a polarization signal that was 50% of the maximum value obtained from direct FP measurements. The protein/8-Fluo-cGMP mixture was incubated with varying concentrations of unlabeled cGMP or cAMP (Biolog Life Science Institute, Bremen, Germany). FP signals were detected as indicated above. Data were analyzed with GraphPad Prism 5.03 by plotting the polarization signal in mPol against the logarithm of the cyclic nucleotide concentration. The EC_50_ values were calculated from sigmoidal dose-response curves.

### Microfluidic mobility-shift assay

Kinase activity was determined using a microfluidic mobility-shift assay on a Caliper DeskTop Profiler (Caliper Life Sciences, PerkinElmer). *Pf*PKG was incubated for 2 hr at 25°C in a 384 well assay plate (Corning, low volume, non-binding surface) in 20 μl buffer (20 mM MOPS pH 7.0, 150 mM NaCl, 0.1 mg/ml BSA, 1 mM DTT, 0.05% L-31, 10 μM FITC-PKS, 990 μM PKS (amino acid sequence: GRTGRRNSI), 1 mM ATP, 10 mM MgCl_2_) and various concentrations of cGMP (3 nM—5 mM), respectively. Reaction mixtures without cyclic nucleotide were used as controls. For electrophoretic separation of substrate and product, a ProfilerPro LabChip (4-sipper mode; Caliper Life Sciences, PerkinElmer) was used under the following conditions: downstream voltage-500 V, upstream voltage-1,900 V with a screening pressure of-1.3 psi. Substrate conversion was plotted against the logarithmic cyclic nucleotide concentration and activation constants (*K*_*a*_) were calculated from sigmoidal dose-response curves employing GraphPad Prism 5.03.

### *Plasmodium falciparum* culture, transfection, and synchronization

*P*. *falciparum* lines 3D7/attB [22], 3D7/PfPKGT618Q [6], and 3D7/PfPKG-HA [35] were cultured according to standard procedures [36] in human A+ erythrocytes (National Blood Transfusion Service, UK) and RPMI 1640 medium supplemented with 0.5% Albumax type II (Lifetech) and 5 nM WR99210 (Jacobus Pharmaceuticals, New Jersey).

3D7/attB ring stage parasites were co-transfected with the pPfPKG/attP expression plasmid and the bxb1 integrase plasmid (pINT) to facilitate recombination of the attP plasmid with the attB site integrated into the cg6 pseudogene locus as described [22]. The following drugs were applied 24 hours post transfection for drug selection: G418 (Sigma, 250 μg/ml) for five days to select for the presence of pINT, and blasticidin S HCl (Sigma, 5 μg/ml) to select for the presence of the pPfPKG/attP plasmid. Blasticidin-resistant parasite cultures were established approximately three weeks post transfection. Transfectant cultures were maintained on blasticidin and WR99210 pressure.

Parasite synchronization was achieved by repeated sorbitol treatments. Highly synchronous parasites were obtained by magnet purification of mature schizonts (MACS, Miltenyi Biotech) followed by the addition of fresh erythrocytes and a sorbitol treatment three to five hours later.

### Construction of *P*. *falciparum* transfection constructs

The R484A single and R484A/Q532A/D533A triple mutations were introduced into pTRC-*Pf*PKGT618Q [6] by site-directed mutagenesis using the QuickChange XL kit (Agilent Technologies) and the following primers: R484A sense; GTACCTTAGGAAAGAATGATTACTTTGGTGAAGCAGCTTTATTATATGATGAAC, R484A anti-sense; GTTCATCATATAATAAAGCTGCTTCACCAAAGTAATCAT TCTTTCCTAAGGTAC, Q532A/D533A sense; AGCACATTTGGAAGAAAGAATAA AAATGGCAGCTACTAAAGTAGAAATGGATGAACTAGA, and Q532A/D533A anti-sense; TCTAGTTCATCCATTTCTACTTTAGTAGCTGCCATTTTTATTCTTTC TTCCAAATGTGCT. Introduction of the desired mutations was confirmed by sequencing. The transfection vector pDCattP-Ama1-PKGT618Q-3xHA was constructed from pDC2-cam-mRFP-Vps4wt-bsd-attP [37,38]. The expression cassette was excised with *Bam*HI and *Hpa*I and replaced with a *Bgl*II-*Pst*I-*Not*I-*Spe*I-*Bam*HI linker (annealed oligos AGATCTCTGCAGGCGGCCGCACTAGTG and GATCCACTAGTGCGGCCGCCTGCAGAGATCT resulting in pDCattP-linkerI. A fragment containing a triple haemagglutinin (3xHA) tag and the PbDT 3’UTR was amplified from pHH1-PKG-HA-3’UTRp.falc [35] with primers 3xHA+PKG 3'UTR fwd *Spe*I gatcACTAGTTTACGATGTTCCTGACTATGC and 3xHA+PKG 3'UTR rev *Bam*HI gatcGGATCCCCAACACCATTCAGAGGTTTA and cloned into the *Bam*HI and SpeI sites of pDCattP-linkerI to make pDCattP-3xHA. The AMA1 promoter/5’UTR was amplified from pAMA1–5'-Sub2-HA3Rep20 [39] (a kind gift from Mike Blackman) using primers ama1 5’ fwd *Bam*HI agtcGGATCCCAAAGAAGAAGCTCAGAGATTGCA and ama1 5’ rev *Not*I-*Pst*I ttttGCGGCCGCttttCTGCAGTCGAGGGCCCTTTTGTACAAT, and cloned into pDCattP-3xHA via *Bam*HI and *Not*I sites to make pDCattP-ama1 5’-3xHA. A *Bgl*II/*Not*I/*Spe*I/*Xho*I/*Pst*I linker was then cloned into the *Bam*HI and *Pst*I site of pDCattP-ama1 5’-3xHA using annealed oligos *Bgl*II/*Not*I/*Spe*I/*Xho*I/*Pst*I linker F GATCTGCGGCCGCACTAGTctcgagCTGCA and *Bgl*II/*Not*I/*Spe*I/*Xho*I/*Pst*I linker R GctcgagACTAGTGCGGCCGCA to yield pDCattP-ama15’linkerII-3xHA. The resulting plasmid was then cut with *Pst*I and *Spe*I in the linker and full-length PKGT618Q PCR products amplified from pTRC-*Pf*PKGT618Q (with or without additional mutations) with primers PKG-fwd-*Pst*I agctCTGCAGATGGAAGAAGATGATAATCTAAAAA AAG and PKG-rev-*Nhe*I agctGCTAGCAAAATCTATGTCCCAGTTGTCTTC introduced. The PKGT618Q-3xHA gene in the final constructs was sequenced again.

### Integration PCR

To confirm efficient integration of the plasmids into the cg6/attB locus, a comprehensive PCR analysis was performed on genomic DNA from transgenic parasites using the following primers: P1 (cg6 5’ coding) CCAGGATCCAAAAGAAGGAGGAGG, P2 (blasticidin deaminase 5’ coding) ATGCATGCCAAGCCTTTGTCTCAAG, P3 (hrp2 3’UTR reverse) TATGTATTTTTTTTGTAATTTCTGTG, P4 (PcDT 5’UTR forward) ATACACTTTCCTTTTTTGTCACT, P5 (M13 reverse (-48)) AGCGGATAACAATTTCACACAGGA, P6 (PF3D7_1222600 forward) GTGAATAATGCAAATCAAACTG, P7 (PF3D7_1222600 reverse) AATATTCCTGTTGTTTCCCCCTTTGTGG. All PCR amplifications were run for 30 cycles using Phusion High-Fidelity DNA polymerase (New England Biolabs) and the manufacturer’s recommended profiles.

### Extraction of PKG from *P*. *falciparum* schizonts, SDS-PAGE and immuno-blotting

Schizont stage parasite cultures were harvested by centrifugation, erythrocytes lysed with 0.15% saponin in PBS and the parasite pellet washed twice in PBS. Parasites were resuspended in ice-cold lysis buffer (10 mM Tris-HCl, 150 mM NaCl, 0.5 mM EDTA, 0.5% NP40, pH 7.5) and incubated on ice for 20 minutes. All buffers were supplemented with complete protease inhibitor cocktail (Roche). The samples were centrifuged at 16000 g for 20 minutes at 4°C and the supernatant assayed by SDS-PAGE/western blot. Proteins were resolved on a NuPAGE Novex 4–12% Bis-Tris gel (Lifetech) and blotted on to nitrocellulose membrane. The membrane was blocked with 5% milk in PBS and probed with the following antibodies: anti-human PKG 1:2500 (rabbit, Enzo Life Sciences); anti-HA 3F10 1:5000 (rat, Roche Diagnostics); anti *Pf*GAPDH 1:15000 (mouse, obtained from Claudia Daubenberger, Swiss Tropical and Public Health Institute). HRP-coupled anti-mouse, rabbit, and rat secondary antibodies (DAKO) were used at 1:6000.

### Schizont accumulation assay and growth assays

*P*. *falciparum* cultures were synchronised by repeated sorbitol lysis, then either compound 2 (1μM) or DMSO was added to late trophozoites / early schizonts and cultures harvested 16h later to count newly formed ring stage parasites. Cells were fixed in 4% formaldehyde/0.1% glutaraldehyde in PBS for 30 minutes at room temperature, stained with SYBR green (Lifetech) diluted 1:10000 in PBS for 30 minutes, washed with PBS and analysed on a FACSCalibur cell analyser (Becton Dickinson). FACS data were analysed using FlowJo software. For growth assays, starting parasitaemia was 0.2% ring stages and cultured in presence or absence of compound 2 (1 μM). Samples were taken every 48h and fixed, stained, and analyzed as described above.

### Coordinates

Atomic coordinates and structure factors of the *Pf*CNB-D:cGMP complex and apo structures have been deposited in the Protein Data Bank (http://www.pdb.org) under accession numbers 4OFG and 4OFF.

## Supporting Information

S1 Fig Sequence alignment between *Pf*CNB domains. Conserved residues are shaded in yellow (100% of identity), in green (75%, of identity), and in gray (over 50% of identity). The capping motif forming residues are typed in red. The key cGMP contact residues in *Pf*CNB-D are marked with arrows.
(EPS)

S2 Fig The crystal contacts at the C-terminus of the apo structure. The αC helix from one molecule (Molecule A’) makes several contacts with the PBC region of a neighboring molecule in the crystal lattice (Molecule A). Molecule A is shown with its N3A motif colored in deep teal, the β barrel region in dark wheat, and the αB-C helices in red. Molecule A’ is shown with its PBC colored in yellow and the rest in gray. The sulfate ions are colored with carbon in yellow and oxygen in red. A zoomed in view of the crystal contact is shown on the right. The surface of Molecule A is rendered in mesh and that of Molecule A’ in transparent surface.
(EPS)

S3 Fig A stereo view of the cGMP pocket. The bound cGMP and cGMP pocket residues are shown in stick with electron density. A |*Fo|-|Fc|* omit map is contoured at σ = 1.0. The cGMP interacting residues and cGMP are labeled and colored by atom type (carbon, white; nitrogen, blue; oxygen, red; phosphorus, orange) except for the carbon atoms. The carbon atoms belonging to the PBC are colored in yellow, the β4-β5 strands in dark gray, αC helix in red, and cGMP in white.
(EPS)

S4 Fig A hydrophobic network seen in the apo state and the PBC adapting a compact conformation upon cGMP binding. (A) A hydrophobic core formed in the apo structure. Individual hydrophobic core forming residues are shown with transparent surface. The conserved hydrophobic hinge residues are marked with a dotted line. (B) Structures of the apo and *Pf*CNB-D:cGMP complex are aligned at the PBC. The hydrogen bond between cGMP and A484 is shown with a dotted line.
(EPS)

S5 Fig A stereo-view of a hydrogen-bonding network at the α3_10_-αA loop and the αB-αC loop. An extensive hydrogen-bonding network is formed between the α3_10_-αA and the αB-αC loops mediated through ordered water molecules. The secondary structures are shown with yellow coil. The residues that participated in the network are shown with the following color scheme: side chain carbon, black; oxygen, red; nitrogen, blue. The ordered water molecules are shown as blue spheres.
(EPS)

S6 Fig The elution profiles of wild-type (WT), R484A, Q532A, D533A and R484A/Q532A/D533A *Pf*PKG (1–853) on a size-exclusion column (Superdex 200) showing with SDS–PAGE gel of elution fraction samples. The wild type and mutant proteins elute at the same volume from a size exclusion column, suggesting that their stokes radius (thus overall conformations) are very similar.
(EPS)

S7 Fig Efficient integration of transfected attP plasmids into the cg6/attB locus. Plasmids pINT and pDCattP_PKG-HA_T618Q_, pDCattP_PKG-_HAT618Q/R484A_, or pDCattP_PKG-HA_T618Q/Triple_, respectively, were co-transfected into the 3D7/attB line. A) Schematic of the plasmids, the parental cg6/attB locus and the modified cg6/attB locus after integration of the pDCattP_PKG-HA plasmid. B) PCR analysis of transfectants to confirm integrase-mediated recombination via attB/attP. PCR templates used were plasmid prep from pDCattP_PKG-HA_T618Q_ (lane 1), genomic DNA from parental 3D7/attB (lane 2), genomic DNA from transfectants 3D7/PKG-HA_T618Q_, 3D7/PKG-HAT_618Q/R484A_, and 3D7/PKG-HA_T618Q/Triple_ (lanes 3 to 5). Primer binding sites and expected amplicon sizes for P1 to P5 are indicated in A). Primer pair P6/P7 amplifies the 3’ end of PF3D7_1222600) and serves as a genomic DNA control.
(EPS)

S8 Fig Transgenic parasites expressing an extra copy of PKG grow at a rate similar to the parental 3D7/attB line. Parasite growth was monitored for 7 days and parasitaemia determined every 48 hours. Parental 3D7/attB line (black diamonds), 3D7/PKG-HA_T618Q_ (light grey squares), 3D7/PKG-HA_T618Q/R484A_ (mid grey triangles), and 3D7/PKG-HA_T618Q/Triple_ (dark grey circles). Data points represent the mean of a single experiment run in triplicates (error bars = standard deviation).
(EPS)

S9 Fig Schematic of the chemical-genetic system used to assess PKG function in *Plasmodium falciparum*. Transgenic parasites harbouring an extra gene copy of HA-tagged *pf*pkg_T618Q_ in the cg6/attB locus express both pkg genes simultaneously. Active wild-type enzyme is produced from the endogenous PfPKG locus while enzyme produced from the cg6/attB locus carries the T618Q substitution conferring resistance to compound 2. Upon addition of compound 2, wild-type enzyme will be inhibited and the parasite depends on the activity of PKG-HA_T618Q_. This system allows us to assess whether a mutation introduced into the PKG-HA_T618Q_ background interferes with kinase activity by asking whether it can complement endogenous PKG function in presence of compound 2.
(EPS)

S1 Table Activation constants of *Pf*PKG _(1–853)_ wild type and mutants. (DOCX)

S2 Table Specific catalytic activity of of *Pf*PKG _(1–853)_ wild type and mutants. (DOCX)
