# Supplementary material for: Crystal Structures of the Carboxyl cGMP Binding Domain of the Plasmodium falciparum cGMP-dependent Protein Kinase Reveal a Novel Capping Triad Crucial for Merozoite Egress
Source: PLoS Pathog. 2015 Feb 3;11(2):e1004639. doi: 10.1371/journal.ppat.1004639 (PMC4412288; doi:10.1371/journal.ppat.1004639)
Supplement: S1 Table — (DOCX) [file ppat.1004639.s010.docx]

**Table S1. Measured activation constants of *Pf*PKG _(1-853)_ wild type and mutants.**

| ***Pf*PKG _(1-853)_** | ***Ka* ± SEM *(n)** | |
| --- | --- | --- |
|  | **cGMP** | **cAMP** |
| Wild Type | 66 ± 2 nM (2) | 14 ± 1 μM (2) |
| R484A | 2.1 ± 0.5 μM (2) | 19 ± 5 μM (2) |
| Q532A | 2.8 ± 0.3 μM (2) | 22 ± 2 μM (2) |
| D533A | 330 ± 10 nM (2) | 11 ± 1 μM (2) |
| R484A/Q532A/D533A | 1.8 ± 0.4 μM (2) | 14 ± 1 μM (2) |
