# Supplementary material for: Crystal Structures of the Carboxyl cGMP Binding Domain of the Plasmodium falciparum cGMP-dependent Protein Kinase Reveal a Novel Capping Triad Crucial for Merozoite Egress
Source: PLoS Pathog. 2015 Feb 3;11(2):e1004639. doi: 10.1371/journal.ppat.1004639 (PMC4412288; doi:10.1371/journal.ppat.1004639)
Supplement: S2 Table — (DOCX) [file ppat.1004639.s011.docx]

**Table S2. Specific catalytic activity of of *Pf*PKG _(1-853)_ wild type and mutants.**

| ***Pf*PKG _(1-853)_** | **Activity [U/mg] ± SEM *(n)** |
| --- | --- |
| Wild Type | 9.3 ± 0.3 (2) |
| R484A | 1.3 ± 0.1 (2) |
| Q532A | 2.6 ± 0.4 (2) |
| D533A | 3.4 ± 0.5 (2) |
| R484A/Q532A/D533A | 0.6 ± 0.0 (2) |
